# The Evidence Base for Wellness Recovery Action Planning (WRAP): A Protocol for a Systematic Literature Review and Meta-Analysis

**DOI:** 10.3390/ijerph182413365

**Published:** 2021-12-19

**Authors:** Michael John Norton, Claire Flynn

**Affiliations:** 1 Office of Mental Health Engagement & Recovery, St. Loman’s Hospital, Palmerstown, D20 HK69 Dublin, Ireland; 2 Adult Continuing Education, The Laurels, University College Cork, College Road, T12 YN60 Cork, Ireland; 3 Mental Health Ireland, Marina House, 11-13 Clarence Street, Dun Laoghaire, A96 E289 Dublin, Ireland; clairef@mentalhealthireland.ie

**Keywords:** mental health, recovery, recovery education, recovery orientation, WRAP

## Abstract

Wellness Recovery Action Planning (WRAP) is a structured recovery education program used internationally for anyone who wishes to create a positive change in their life. It was developed by Mary Ellen Copeland and Jane Winterling in the late 90s, resulting from a search of programs or initiatives that could support their wellness. Since then, many studies have been conducted into the efficacy of the program for those with a mental health/addiction challenge. However, to date, there has been little to no synthesis of the evidence base for WRAP outside of the Copeland Center’s internal reviews. The proposed systematic review and meta-analysis aims to investigate the evidence base for Wellness Recovery Action Planning within a mental health context. To do this, a PRISMA 2020 compliant review is proposed using the PRISMA-P statement as a guide to demonstrate the methodology to be undertaken. It outlines the inclusion/exclusion criteria, search terms, and databases while informing the readership of timescales to complete such actions. Finally, this protocol also addresses issues of bias and quality within included studies. This proposed literature review and meta-analysis will synthesize and examine the evidence base for WRAP using a systematic review methodology. It is hoped that this proposed review will identify gaps in the current literature regarding the program, and provide recommendations that will support the reviewers in their further studies while also strengthening the argument for a whole system evaluation of WRAP within an Irish context.

## 1. Introduction

Mental illness accounts for over 10% of disorders worldwide [1], and the impact of living with a mental health issue can negatively affect all areas of a person’s life [2]. Providing support for a person living with a mental health issue is more than providing medical care, it is also about supporting them towards recovery, and to live a life of meaning and achievement [2,3].

Recovery for those with mental health challenges has traditionally been seen as clinical in nature, resulting in the alleviation or elimination of disease or illness. It is now seen from an individual perspective, and there is an expectation that each person will have a recovery journey that is unique to their experience [3]. However, since the publication of “*A Vision for Change*” [4], mental health services in Ireland have moved towards a recovery-orientated service [5], which is now an accepted philosophy and ethos within mental health [6]. Recovery from this perspective refers to living a hopeful, contributing, and fulfilling life of one’s choosing regardless of the presence of mental ill health [7]. This idea led to the widespread understanding that those with a mental health challenge, and indeed the family that supports them, have a recovery process to follow by which one learns to live with the symptoms of distress, and build a life of their own choosing [8]. Wellness is defined as “*the quality or state of being in good health especially as an actively sought goal*” [9]. A recovery journey is in itself a journey to wellness [10]. One such method of doing this is through the development of a Wellness Recovery Action Plan (WRAP).

WRAP is an acronym that refers to a structured wellness program for individuals who wish to make positive changes in their life towards recovery, including their own self-defined goals. The program was conceptualized by Mary Ellen Copeland and Jane Winterling in 1997. To deal with increased demand for this program, the Copeland Center was created and became a governing body for WRAP in 2003 [11]. The program is now utilized in many settings, including within mental health services, where it acts as a scaffold for service users to build their recovery on. Currently, within an Irish context, mental health services and charitable organizations have been offering this evidence-based program for many years [5,11,12,13,14,15,16]. WRAP has been evolving in Ireland since its introduction in the late 1990s. Although WRAP has been delivered throughout the island of Ireland, there is still little peer-reviewed evidence to examine the impact of WRAP to those who use it in an Irish context.

### 1.1. Rationale for Proposed Systematic Review and Meta-Analysis

WRAP has been well researched both on a national and international level [17,18]. However, there is little evidence that examines its application in other countries and cultures. Additionally, little to no synthesis has been completed thus far into the evidence base of WRAP outside of the Copeland Center’s own internal reviews. From a search of the available literature, only two systematic reviews appeared, one was a systematic review and meta-analysis of randomized controlled trials into the effectiveness of the program on a service user’s clinical recovery outcomes [19]. The other was a systematic review of qualitative data collected by included studies which examined the effects of WRAP on service users, while also examining the quality of qualitative literature available [20]. Although scholars are beginning to synthesis evidence into this initiative, no such review has been carried out thus far on all literature typologies into WRAP. There may be many reasons for this; however, the most plausible rationale for this is due to the fact that WRAP is still in its infancy, as it was only established in 1997, and as such, no such synthesis could be justified up until this point. Added to this, both reviewers are involved in a project within the Irish mental health services to standardize the delivery of WRAP, and as such, a complete synthesis of the evidence base will prove useful to support the work of both reviewers, particularly in identifying if an up-to-date evaluation of current practices as they relate to WRAP is warranted, and also in identifying what methodological and theoretical approaches the reviewers need to be cognizant of as they progress this project within an Irish mental health care context. Therefore, to access this knowledge, and to combat this paucity of complete synthesis currently evident within the peer reviewed literature, the proposed systematic review and meta-analysis will explore the evidence base for WRAP using a systematic process of reviewing peer reviewed literature available into the program on both a national and international basis.

### 1.2. Objectives

The proposed systematic review and meta-analysis aims to examine the evidence base for a recovery educational program: WRAP within the available, peer reviewed literature. To achieve this, the proposed systematic review and meta-analysis will aim to achieve the following objectives:

- To examine the utilization of WRAP in different cultures and health services internationally.
- To identify if an evaluation of WRAP delivery in Irish services is required, given the changes in services to recovery orientation.
- To identify gaps in the current literature base as it pertains to the delivery of WRAP.
- To make recommendations for further study and research with WRAP, particularly from an Irish context.

## 2. Methods and Analysis

This proposed systematic review and meta-analysis will utilize the newly updated Preferred Reporting Items for Systematic reviews and Meta-Analysis (PRISMA) standardized reporting guidelines [21]. However, the reviewers acknowledge that in order to conform with best practice in reporting for systematic reviews, a protocol should be created and published prior to commencement of the actual review [22]. Therefore, this protocol was created. Additionally, to support best practice as guided by PRISMA, this protocol will comply with the PRISMA-P guidelines [23], the results of which are reported in Table A1. This protocol was registered with OSF repositories on the 18th November 2021. Any amendments made to this systematic review will be noted and uploaded to the registry where it is freely available for viewing as required.

### 2.1. Eligibility Criteria

As this review seeks to understand the entire literature base for WRAP, no research question was developed. However, the PICO framework [24,25] was utilized to support the breakdown of our objectives into tangible terms that could be used to search the databases for appropriate papers. To support the selection of all relevant articles for this review, an inclusion/exclusion criterion was created (Table 1).

As stipulated in Table 1, a search range dating back 10 years from the search date will be enforced so that the latest evidence, as it pertains to WRAP, can be collected. Any peer reviewed research study will be included. Any articles that are not written in the English language will be excluded. For the purposes of this review, the research team [MJN, CF] are only interested in articles discussing individual WRAP in mental health services, so as to align with the objectives of both this protocol and proposed systematic review, and the meta-analysis.

### 2.2. Information Sources

The following search will be undertaken to explore the published peer reviewed literature into WRAP within a mental health context. The search will be undertaken using the following databases: CINAHL, JSTOR, OVID SP, PsycARTICLES, PsycINFO, PubMed, Science Direct, Web of Science, Wiley Online Library, and EBSCHO host. To ensure that all available peer reviewed documents are collected, a reference search will also be undertaken on included articles.

### 2.3. Search Strategy

The following search terms will be used in the proposed systematic review and meta-analysis: “wellness recovery action plan” OR “WRAP” OR “wellness recovery action planning” OR “wellness plans” OR “advanced directives” OR “crisis plans”

AND

“online” OR “internet” OR “in-person” OR “face-to-face”

AND

“experiences” OR “perceptions” OR “thoughts” OR “opinions” OR “views” OR “feelings” OR “judgements”

AND

“mental” OR “psychiatric” OR “mental illness” OR “psychiatric disorder” OR “mental ill health” OR “mental health” OR “mental wellness” OR “recovery” OR “rehabilitation.”

### 2.4. Study Records

**Figure 1:**
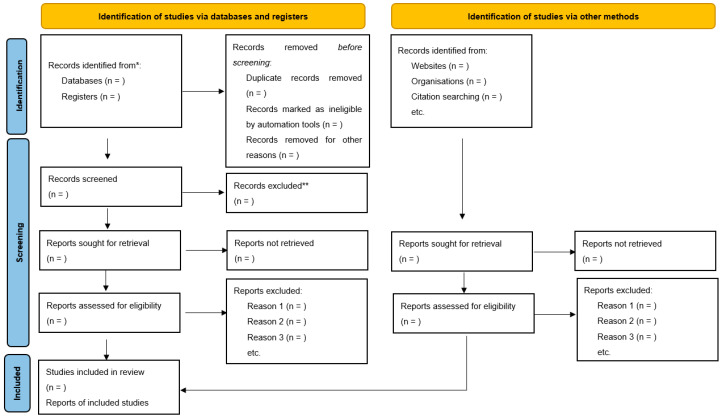
PRISMA 2020 flow diagram.

This systematic review and meta-analysis will employ three rounds of searching. Round one will focus on the title of papers. Any papers that have WRAP or any of its synonyms (see above) in its title will be extracted and saved in the round one folder. Once this is complete, a second round will occur. This comprises two processes. Firstly, duplicates will be removed. After which, the abstracts of saved papers will be read, and using the above inclusion/exclusion criteria (Table 1), papers will be methodically selected. Round three then occurs, where the full text of articles will be read, following which, articles may be excluded based on the inclusion/exclusion criteria. To ensure all necessary papers were collected, the reference list of final included papers will be searched, and such papers will undergo the same methodical inclusion process. This process will be demonstrated using the updated PRISMA flow diagram (Figure 1). Both authors will undertake a search of the literature, and be involved in all rounds of searching.

This part of the process will begin on the 1st February 2022. Round three searching is expected to be completed by the 31st March 2022. After which, relevant information from included papers will be extracted and charted in a comparative table to demonstrate critical thinking. The following information will be extracted for this comparative appraisal table:

- Author’s surname
- Year of publication
- Country
- Aim of study
- Study design—qualitative, quantitative or mixed method
- Methodological orientation—if known
- Data collection methods
- Sample and sample size
- Study findings/results
- Theoretical framework—if known

This process is expected to take the first half of April 2022 to complete (1–15 April 2022). Both authors will be involved in creating the comparative appraisal table. 

### 2.5. Risk of Bias in Individual Studies

Risk of bias within individual studies will also be assessed as part of the quality appraisal process. Bias will be assessed under the following headings: performance, selection, and attrition bias. To demonstrate the outcome of such assessments, McGuiness and Higgins [26] Robvis web-based application will be utilized. This tool was first developed in 2019 to support reviewers in both the assessment and visualization of risk of bias within individual studies [26]. It is a well-trusted method of demonstrating such biases in a timely and efficient manner, and will be utilized for all article types, including qualitative and mixed method studies. This was successfully used in one author’s previous work [22] to demonstrate the original authors’ biases in their reporting of data. Both authors will assess the risk of bias separately. However, a meeting will then be held to discuss such results, and any disagreements will be discussed until consensus is reached.

### 2.6. Assessing the Quality of Evidence

As part of this review, the quality of evidence will also be assessed. Here, variations of the Critical Appraisal Skills Program (CASP) Tool [27,28,29] and the Mixed Method Appraisal Tool (MMAT) [30] will be used to assess quality, as this review will include studies with multiple methodologies.

### 2.7. Data Synthesis

This review will employ Brawn and Clarke’s [31] thematic analysis to analyze and synthesize qualitative data. This will involve the reviewers becoming familiar with the data presented in included studies through many readings of these papers. From which, initial codes will be generated, resulting in themes. These will be revised, adjusted, and refined by both authors, and will be supported by both authors’ subjectivities and pre-conceived ideas about the research question under inquiry, and will be further supported through reflections.

As this proposed review is including quantitative and mixed method studies exploring WRAP, a meta-analysis may be necessary, dependent on the number of such studies in the final inclusion list. If such data are included after the screening process is complete, a meta-analysis will be conducted on the data using a forest plot approach, as stipulated by Neyeloff and colleagues [32]. A meta-analysis is a type of review which focuses on synthesizing quantitative data from included independent studies [32,33]. It is best illustrated through a forest plot, which consists of a figure within the findings of the proposed review which demonstrates the overall pattern of results from all included studies by show pointing estimates arising from these studies [32,34]. It is particularly useful when included studies conform to the same conditions and/or treatments [32].

### 2.8. Patient and Public Involvement

This systematic review and meta-analysis protocol did not involve individual patients/service users or public agencies.

## 3. Discussion

This proposed systematic review and meta-analysis will use the PRISMA 2020 statement to guide the review in examining the evidence base for the recovery education program, WRAP. In doing so it will also examine the use of the program in other cultures and mental health services internationally. This may be an important finding which could support the rationale for representatives of the Copeland Center to expand their current work remit to include cultures/services that do not utilize the program. In terms of an evaluation in an Irish context, the reviewers are aware of two evaluations that have occurred previously within an Irish context by Agnes Higgins and colleagues [17], and Horan and Fox [18]. However, these evaluations are outdated and do not take in WRAP delivered using the most up-to-date curriculum available. As such, this review is necessary for the reviewers to determine if a new evaluation is needed, as well as what should be examined within such an evaluation in the future. This review will also make recommendations for future research into this recovery educational program that will support the reviewers in any future research undertaken. For example, this proposed review will add clarity to the peer reviewed academic literature in regards to the quality of evidence available in the literature, while also potentially identifying the presence or lack of presence of theory as it relates to WRAP, both of which are findings that could initiate further investigation in order to improve the literature, and relate it more so to theory. Finally, this proposed review could have potential positive applications to society at large, as it will identify the best available evidence as it relates to WRAP. This is achieved through only including studies that are less than 10 years old at the time of the search. This best available evidence will then be documented and displayed to the audience through the write up of this review. Resulting from this process, it is hoped that readers will: 1. Take up WRAP in their own lives; 2. Realize the evidence base of WRAP; and 3. Practice and deliver WRAP as per the values and ethics of the program while adhering to the fidelity of WRAP. All of these steps will benefit those utilizing the program in their own lives. 

### Strenghts and Limitations of the Proposed Review

As with all systematic reviews and meta-analysis, there are some strengths and weaknesses to this proposed review. In terms of strengths, this review is possibly the first to systematically synthesize all study types as they relate to WRAP. The review will utilize the most up-to-date PRISMA guidelines to guide and structure this systematic review and meta-analysis. It will use evidence-based tools, such as CASP and MMAT, to measure quality of all included papers, and Robvis to measure and visualize the potential biases of papers included in the review. Finally, the review will also focus on papers from the last 10 years. Though this is good practice in terms of evidence-based research, it is also important to ensure that we collect data from sources who kept to the fidelity of the program.

There are also several limitations to this proposed review. Firstly, as this proposed paper will be a systematic review, it will not capture the pure breadth of literature available (peer reviewed and gray) on the program. Although included studies will undergo a screening process, there is still a possibility that the included papers may be: 1. Of poor quality; and 2. From only a select region, which may impact the generalizability and reliability of the review results.

## Figures and Tables

**Table 1:** Inclusion/exclusion criteria.

Inclusion	Exclusion
Peer Reviewed Qualitative, Quantitative, and Mixed Method Articles	Editorials, Media Articles, Opinion Pieces, Gray Literature (Published Study), Systematic Reviews, Literature Reviews, Rapid Reviews, Meta-Analysis, Meta-Synthesis
	Dissertations
English Language	
Articles within Past 10 Years	
WRAP in Adulthood	WRAP in Children/Adolescence
WRAP for Mental Health	WRAP for Addictions/Physical Health, Intellectual Disability/Dual Diagnosis
Individual WRAP	Family WRAP

## Data Availability

Not applicable.

## Appendix A. PRISMA-P Checklist

**Table A1:** PRISMA-P (Preferred Reporting Items for Systematic review and Meta-Analysis Protocols) 2015 checklist: recommended items to address in a systematic review protocol *.

Section and Topic	Item No	Checklist Item	Achieved?
**Administrative Information**			
Title:			
Identification	1a	Identify the report as a protocol of a systematic review	YES
Update	1b	If the protocol is for an update of a previous systematic review, identify as such	N/A
Registration	2	If registered, provide the name of the registry (such as PROSPERO) and registration number	YES
Authors:			
Contact	3a	Provide name, institutional affiliation, e-mail address of all protocol authors; provide physical mailing address of corresponding author	YES
Contributions	3b	Describe contributions of protocol authors, and identify the guarantor of the review	YES
Amendments	4	If the protocol represents an amendment of a previously completed or published protocol, identify as such, and list changes; otherwise, state plan for documenting important protocol amendments	N/A
Support:			
Sources	5a	Indicate sources of financial or other support for the review	N/A
Sponsor	5b	Provide name for the review funder and/or sponsor	N/A
Role of sponsor or funder	5c	Describe roles of funder(s), sponsor(s), and/or institution(s), if any, in developing the protocol	N/A
**Introduction**			
Rationale	6	Describe the rationale for the review in the context of what is already known	YES
Objectives	7	Provide an explicit statement of the question(s) the review will address with reference to participants, interventions, comparators, and outcomes (PICO)	YES
**Methods**			
Eligibility criteria	8	Specify the study characteristics (such as PICO, study design, setting, time frame) and report characteristics (such as years considered, language, publication status) to be used as criteria for eligibility for the review	YES
Information sources	9	Describe all intended information sources (such as electronic databases, contact with study authors, trial registers or other gray literature sources) with planned dates of coverage	YES
Search strategy	10	Present draft of search strategy to be used for at least one electronic database, including planned limits, such that it could be repeated	YES
Study records:			
Data management	11a	Describe the mechanism(s) that will be used to manage records and data throughout the review	YES
Selection process	11b	State the process that will be used for selecting studies (such as two independent reviewers) through each phase of the review (that is, screening, eligibility, and inclusion in meta-analysis)	YES
Data collection process	11c	Describe planned method of extracting data from reports (such as piloting forms, done independently, in duplicate), any processes for obtaining and confirming data from investigators	YES
Data items	12	List and define all variables for which data will be sought (such as PICO items, funding sources), any pre-planned data assumptions and simplifications	YES
Outcomes and prioritization	13	List and define all outcomes for which data will be sought, including prioritization of main and additional outcomes, with rationale	N/A
Risk of bias in individual studies	14	Describe anticipated methods for assessing risk of bias of individual studies, including whether this will be done at the outcome or study level, or both; state how this information will be used in data synthesis	YES
Data synthesis	15a	Describe criteria under which study data will be quantitatively synthesised	YES
	15b	If data are appropriate for quantitative synthesis, describe planned summary measures, methods of handling data, and methods of combining data from studies, including any planned exploration of consistency (such as I^2^, Kendall’s τ)	YES
	15c	Describe any proposed additional analyses (such as sensitivity or subgroup analyses, meta-regression)	N/A
	15d	If quantitative synthesis is not appropriate, describe the type of summary planned	YES
Meta-bias(es)	16	Specify any planned assessment of meta-bias(es) (such as publication bias across studies, selective reporting within studies)	YES
Confidence in cumulative evidence	17	Describe how the strength of the body of evidence will be assessed (such as GRADE)	N/A

*** It is strongly recommended that this checklist be read in conjunction with the PRISMA-P Explanation and Elaboration (cite when available) for important clarification on the items. Amendments to a review protocol should be tracked and dated. The copyright for PRISMA-P (including checklist) is held by the PRISMA-P Group, and is distributed under a Creative Commons Attribution License 4.0.**
